# Changes in coagulation markers in children with *Mycoplasma pneumoniae* pneumonia and their predictive value for *Mycoplasma* severity

**DOI:** 10.1186/s13052-023-01545-1

**Published:** 2023-10-20

**Authors:** Yong-tao Li, Ju Zhang, Meng-zhu Wang, Yu-Mei Ma, Ke Zhi, Fu-Li Dai, Shu-jun Li

**Affiliations:** 1 Department of Pediatrics, Luoyang Maternal and Child Health Hospital, No. 206 of Tongqu Road, 471000 Luoyang, Henan province China; 2 Department of Pediatrics, The First Affiliated Hospital of Xinxiang Medical University, No. 88 of Jiankangroad, 453100 Weihui, Henan province China

**Keywords:** Coagulation, *Mycoplasma pneumoniae* pneumonia, Predictive value, Children

## Abstract

**Background:**

This study investigates the correlation between coagulation levels and the severity of *Mycoplasma* pneumoniae pneumonia (MPP) in children. In addition, the study analyses the predictive value of coagulation abnormalities in MPP combined with necrotising pneumonia (NP).

**Methods:**

A total of 170 children with MPP who underwent treatment between June 2021 and February 2022 were selected for this study. The study population was divided into groups according to the severity of the disease to compare differences in the incidence of coagulation abnormalities between the groups. The participants were also divided into groups according to imaging manifestations to compare the differences in coagulation function among the different groups. All data information was processed for statistical analysis using SPSS Statistics 25.0 and GraphPad Prism 7.0 statistical analysis software.

**Results:**

The incidence of coagulation abnormalities in the children in the severe MPP (SMPP) group was significantly higher than that in the normal MPP (NMPP) group (*P* < 0.05). The multi-factor logistic regression analysis revealed that the D-dimer level is an independent risk factor for the development of NP in SMPP (*P* < 0.05). The receiver operating characteristic curve analysis revealed statistically significant differences (*P <* 0.05) in D-dimer, fibrinogen degeneration products (FDP), neutrophils, lactate dehydrogenase and serum ferritin for predicting SMPP combined with NP. Bronchoscopic manifestations of coagulation indicators (D-dimer and FDP levels) were significantly higher in the mucus plug group than in the non-mucus plug group, while the activated partial thromboplastin time levels were lower in the former than in the latter (*P <* 0.05).

**Conclusion:**

The degree of elevated D-dimer and FDP levels was positively correlated with the severity of MPP, with elevated serum D-dimer levels (> 3.705 mg/L) serving as an independent predictor of MPP combined with NP in children.

## Introduction

*Mycoplasma pneumoniae* pneumonia (MPP) is a common cause of community-acquired pneumonia in children, accounting for approximately 40% of pneumonia cases in children over 5 years of age. [1, 2] It may be more prevalent during epidemic periods. [3].

Although MPP is generally considered to be a self-limiting disease, some children, especially those of an older age, may progress to severe MPP (SMPP) or refractory MPP (RMPP). The causes may be related to direct pathogen damage, MP resistance, abnormal immune inflammatory responses and mixed infections, [4] but the exact mechanisms are unclear. Severe MPP can also be combined with pulmonary complications, such as pleural effusion, atelectasis and necrotising pneumonia (NP), while in severe cases, respiratory failure and hypoxaemia may occur, which can be fatal. [5] Distant sequelae may include occlusive bronchitis, permanent pulmonary atelectasis and bronchiectasis.

In addition to causing lung damage, MPP can lead to a range of extra-pulmonary complications, including liver damage, encephalitis of the central nervous system, haemolytic anaemia and thrombosis. Early recognition of severe cases is thus essential.

Recent studies have shown that coagulation abnormalities are not uncommon in children with MPP. The specific mechanism of abnormal coagulation function occurring in MP infection is unclear but may be related to MP inducing massive synthesis and secretion of a series of cytokines, such as interleukins, tumour necrosis factors and chemokines, causing local vascular damage and resulting in the accumulation of metabolites at this site, causing a vascular blockage. [6] Among these metabolites is D-dimer, a specific marker of the fibrinolytic system that also serves as an indicator for monitoring inflammation and serious infections. [7] It was also found that abnormal coagulation could be involved in the development of SMPP or RMPP and may be closely related to the development and prognosis of its complications; [8, 9] however, there are relatively few studies on the correlation between coagulation indicators and the severity of MPP in children. [10].

Our study of coagulation function in children with MPP, its correlation with disease severity and its predictive value for NP can assist in the early identification of severe cases, provide reliable data to support early and aggressive treatment and help improve the outcome and prognosis. The difference between coagulation and different MPPs was also explored in greater depth using different imaging and bronchoscopic presentations.

## Patients and methods

### Patients

A total of 170 children with MPP who received treatment at Luoyang Maternal and Child Health Hospital between June 2021 and February 2022 were consecutively selected as the study population. The patients were diagnosed with MPP according to the ‘Expert Consensus on the Diagnosis and Management of *Mycoplasma pneumoniae* Pneumonia in Children’ (2015 version), [11] as developed by the Respiratory Group of the Paediatrics Branch of the Chinese Medical Association. Briefly, the diagnostic criteria of MPP are as follows: (1) cough, with or without fever and other respiratory manifestations, and double lung auscultation can be heard along with dry and wet rales or signs of solid changes; (2) imaging suggests that the lungs present lobar infiltration or staged solid changes, lobular punctate infiltration or interstitial changes; and (3) MP infection: a single serum MP antibody titre of > 1:160, or twice 4-fold rise in titer in serum MP antibody, or a positive MP-polymerase chain reaction in a throat swab or alveolar lavage fluid.

For the present study, the diagnostic criteria for MPP combined with NP were observed as follows: (1) Previous lung imaging was normal, and, based on a definite diagnosis of MPP, an early computed tomography (CT) scan of the lungs suggested solid lung changes or combined pleural effusion. (2) As the disease progressed, liquefied necrosis of the lung tissue was observed in the original solid lung area, with multiple small, thin-walled cavities filled with gas or fluid; multiple small cavities may also have fused to form larger cavities, [12] which may also have included liquid–gas planes.

There are no clear diagnostic criteria for SMPP, but reference can be made to the ‘Code of Practice for the Treatment of Community-Acquired Pneumonia in Children’ (2019), [13] the ‘Expert Consensus on the Combined Diagnosis and Treatment of *Mycoplasma pneumoniae* Pneumonia in Children with Chinese and Western Medicine’ (developed in 2017), and the ‘Expert Consensus on the Laboratory Diagnostic Specifications and Clinical Practice of *Mycoplasma pneumoniae* Infection in Children in China’ (2019). The diagnostic criteria for SMPP include: (1) obvious shortness of breath or tachycardia, with or without dyspnea and cyanosis; (2) hypoxemia with pulse oximetry ≤ 0.92 on inspired air; (3) chest imaging showing multi-lobar segmental involvement or involvement of more than two-thirds of the lung; (4) intra-pulmonary complications, such as pleural effusion, atelectasis, necrosis and abscess; and (5) combination of severe damage to other systems (central nervous system infection, heart failure, myocarditis, gastrointestinal bleeding, significant electrolyte/acid-base balance disorders, etc.). Severe MPP can be diagnosed when any of the above is met together with a diagnosis of MPP.

Abnormal coagulation functioning was established using the following: thrombin time (TT) and prothrombin time (PT) prolongation of > 3 s; activated partial thromboplastin time (aPTT) prolongation of > 10 s; and fibrinogen (FIB), FIB degradation products (FDP) and D-dimer levels outside the normal reference range. Any instance where the above indicators were met was considered to reflect abnormal coagulation functioning. All of the patients with NP induced by MP infection were considered to have SMPP.

The exclusion criteria for the current study were as follows: (1) inadequate clinical information available for the child; (2) the condition was in the recovery stage at the time of admission; (3) evidence of co-infection with other pathogenic microorganisms (e.g. *Staphylococcus aureus*, *Streptococcus pneumoniae*, fungi and tuberculosis); (4) presence of other systemic diseases, such as severe congenital heart disease, chronic kidney disease, chronic lung disease, blood disorders and tumours; (5) patients who have received recent anticoagulant treatment or were using anticoagulants for other medical conditions; (6) patients with a recent history of major surgery or serious trauma and blood transfusion; and (7) patients with an immunodeficiency or other diseases that could cause abnormal immune function, as well as those who had recently received immunomodulatory agents.

The study population was divided into the SMPP group and the normal MPP (NMPP) group according to the severity of the disease. Following this, the population was further divided into the NMPP group (Group A, *n* = 83), NMPP combined with solid lung lesions (Group B, *n* = 37), MPP combined with solid lung lesions and pleural effusion (Group C, *n* = 28) and MPP combined with NP (Group D, *n* = 22), based on different imaging presentations. In addition, children with MPP combined with solid lung changes who underwent a bronchoscopy were divided into a mucus plug group and a non-mucus plug group according to their bronchoscopic presentation. This study was approved by the Ethics Committee of Luoyang Maternal and Child Health Hospital (no. KY2022052004.0).

Based on the diagnosis and treatment standard, in the NMPP group, anti-microbial treatment (macrolide anti-microbial drug anti-infection), nebulised inhaled glucocorticoid, sputum and other comprehensive treatments were administered. In the SMPP group, in addition to anti-infection and nebulised inhaled glucocorticoid and sputum treatments, intravenous glucocorticoid treatment was administered according to the severity of the disease. For the children with combined MPP and solid lung lesions, which were in line with the bronchoscopy indications, bronchoscopy and alveolar lavage treatment were performed after obtaining the consent of family members. Moreover, for those with obvious abnormalities in coagulation function, anticoagulant treatment of heparin sodium was given, and the aPTT was monitored.

### Methods

The NMPP group (i.e. the control group) received a combination of treatments based on anti-microbial therapy, according to the treatment norms. The SMPP group (i.e. the observation group) received glucocorticoid therapy in addition to anti-infection and combination therapy, depending on the severity of the disease. [14] Fibreoptic bronchoscopy is generally only performed for patients receiving mechanical ventilation and patients with risk factors for rare microbial infections or complex pneumonia (e.g. immunocompromised or failure of empirical therapy). A bronchoscopy was performed for children who met the indications for the procedure according to the Chinese Guidelines for Paediatric Bendable Bronchoscopy (2018). [15] In addition, those with significant coagulation abnormalities received anticoagulation treatment with sodium heparin and were monitored for aPTT. The incidence of NP induced by MP was 12.35%.

Clinical information that may influence the severity of the disease was collected, including the patient’s general condition, coagulation indicators (TT, PT, aPTT, FIB, FDP), general inflammatory indicators (white blood cell count, neutrophil ratio [NE%], C-reactive protein [CRP], serum ferritin [SF] and serum lactate dehydrogenase), in addition to clinical features/indicators (total length of fever, total length of hospital stay, chest imaging, bronchoscopic manifestations, concurrent tensor and sequelae). All children underwent venous blood sampling within 24 h of admission for routine blood and CRP, coagulation, ferritin, lactate dehydrogenase (LDH) and MP antibody (immunoglobulin [Ig]M). If the first serum MP antibody IgM was negative, blood could be drawn again seven days after the onset of the illness. All patients had acute onset (most within one week of onset; some cases were more than a week old, but the infection was still progressing). All of the children underwent a chest radiograph or CT chest examination on admission according to the ‘Code of Practice for the Management of Community Acquired Pneumonia in Children’ (2019).

The study data were processed for statistical analysis using SPSS Statistics 25.0 (IBM, Chicago, USA) and GraphPad Prism 7.0 statistical analysis software (GraphPad ,California, USA). Relevant information was obtained via a Shapiro–Wilk test, and comparison information across the groups was obtained using the one-way analysis of variance method. Categorical information was expressed as percentages and tested using Chi-square tests. An independent samples *t*-test was conducted if the measures conformed to a normal or approximately normal distribution, using mean ± standard deviation ($\bar x$ ± s ). A non-parametric test (Mann–Whitney) was used for non-normally distributed data, expressed in terms of median and interquartile spacing (M [P25, P75]). Analysis of the predictive value of coagulation indicators in the development of NP in MPP was conducted using logistic regression. Receiver operating characteristic (ROC) curves were created using the variables associated with MPP severity to analyse their sensitivity, specificity and optimal threshold values for predicting MPP combined with NP. All of the above statistical results were considered statistically significant at *P <* 0.05.

## Results

This study included 170 children with MPP, with 102 in the NMPP group and 68 in the SMPP group. The differences in gender composition, age, weight, acute blood leukocyte count, platelet count and platelet distribution width at admission between the NMPP and SMPP groups were not statistically significant (*P* > 0.05). The SMPP group had a higher incidence of total fever duration, total length of hospital stay, CRP, LDH, SF, NE%, mean platelet volume and solid lung changes in the acute phase than the NMPP group, with statistically significant differences (*P <* 0.05) (Table 1).

**Table 1:** Comparison of general demographic data, inflammatory parameters, chest imaging characteristics, and clinical characteristics between theNMPPgroup and the SMPP group

Variables	SMPP group(n = 68)	NMPP group(n = 102)	*p* value
Sex			0.167
Male, n (%)	36(52.94)	43(42.16)	
Female, n (%)	32(47.06)	59(57.84)	
Age(years)	6.4 ± 2.2	6.2 ± 2.7	0.659
Weight(kg)	21.66 ± 5.93	23.24 ± 9.42	0.182
WBC (×10^9^/L)	9.07 ± 3.86	8.50 ± 2.80	0.264
NE (%)	70.49 ± 12.11	59.30 ± 12.11	< 0.001
CRP(mg/L)	30.41(12.32, 55.87)	10.72(5.39, 19.89)	< 0.001
SF (ng/ml)	201.32(101.76, 346.06)	75.82(47.87, 101.92)	< 0.001
LDH(U/L)	442.80(302.05, 640.48)	295.40(262.78, 326.70)	< 0.001
PDW (fl.)	15.80(15.25, 16.30)	15.70(15.40, 15.90)	0.297
PLT (×10^9^/L)	305.32 ± 113.19	295.35 ± 90.26	0.526
MPV (fl.)	9.54 ± 1.10	9.07 ± 0.86	0.005
Lung solids, n (%)	60(88.24)	19(18.63)	< 0.001
Total heat duration(day)	10.79 ± 4.18	6.64 ± 2.54	< 0.001
Total length of hospital stay(day)	17.75 ± 4.33	13.25 ± 2.45	< 0.001

Note: WBC = white blood cell; NE = Neutrophils; CRP = C-reactive protein; SF = serum ferritin; LDH = lactate dehydrogenase; PDW = platelet distribution width; PLT = Platelets; MPV = mean platelet volume;NMPP:Normal Mycoplasma pneumoniae pneumonia; SMPP: Severe Mycoplasma pneumoniae pneumonia

### Comparison of the incidence of coagulation abnormalities and related coagulation indicators across the groups

The incidence of coagulation abnormalities was significantly higher in the SMPP group (70.59%, 48/68) than in the NMPP group (3.92%, 4/102), with a statistically significant difference (*P <* 0.001). The difference between the two groups of children’s coagulation indicators (TT and PT) was not statistically significant (*P* > 0.05). The coagulation indexes (FIB, D-dimer and FDP) were significantly higher in the SMPP than in the NMPP group, whereas the aPTT was lower in the former than in the latter, with statistically significant differences (*P <* 0.05) (Table 2).

**Table 2:** Comparison of coagulation parameters between SMPP group and NMPP group

Coagulation Indicators	SMPP group(n = 68)	NMPP group(n = 102)	*t* /*Z* value	*p* value
TT(s)	17.30 ± 1.71	17.37 ± 1.30	0.288	0.774
PT(s)	13.71 ± 1.33	13.66 ± 1.42	0.241	0.810
APTT(s)	30.27 ± 6.99	33.21 ± 4.41	3.083	0.003
FIB(g/L)	4.24 ± 0.89	3.98 ± 0.74	2.057	0.041
D-Dimer(mg/L)	2.02(0.79, 4.16)	0.38(0.30, 0.57)	8.586*	< 0.001
FDP(µg/mL)	9.33(5.08, 19.70)	2.82(2.08, 3.64)	8.681*	< 0.001

Note: * represents the Z value; TT = thrombin time; PT = prothrombin time; APTT = activated partial thromboplastin time; FIB = fibrinogen; FDP = fibrinogen degradation products; NMPP: Normal Mycoplasma pneumoniae pneumonia; SMPP: Severe Mycoplasma pneumoniae pneumonia

### Comparison of the coagulation function between different imaging groups

**Fig. 1:**
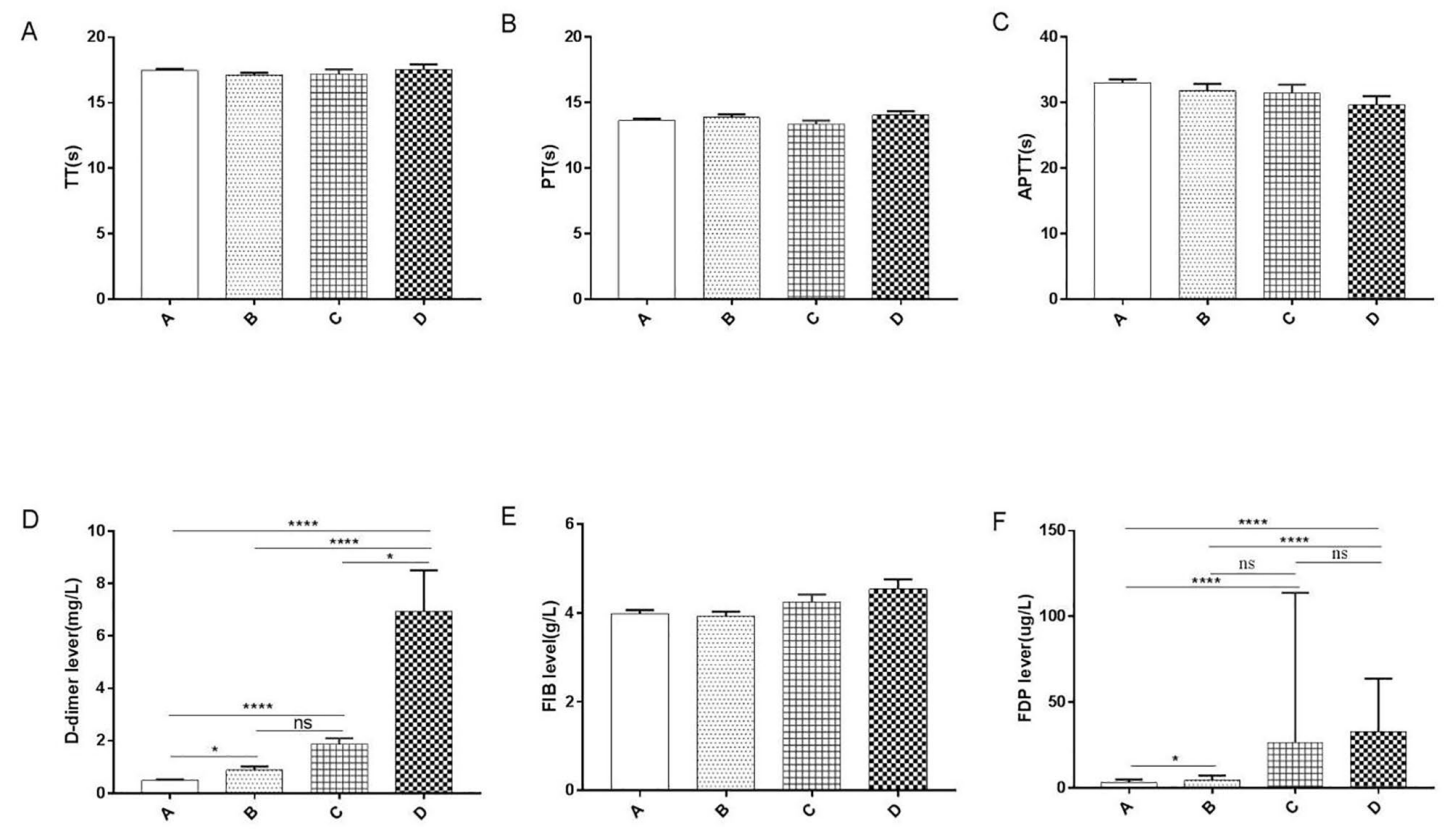
Comparison of coagulation indicators between different imaging groups in MPP. Note: Group A = NMPP group, Group B = NMPP combined with solid lung lesions, Group C = NMPP combined with solid lung lesions and pleural effusion, Group D = NMPP combined with necrotizing pneumonia group

The TT, PT and FIB results were compared between the groups within the simple NMPP group (Group A, 83 cases), the NMPP combined with solid lung lesions group (Group B, 37 cases), the NMPP combined with solid lung lesions and pleural effusion group (Group C, 28 cases) and the NMPP combined with necrotising pneumonia group (Group D, 22 cases); the differences were not statistically significant (*P* > 0.05). The D-dimer, FDP and aPTT levels across the four groups were compared using different imaging presentations: the more severe the chest imaging presentation, the higher the D-dimer and FDP levels and the lower the aPTT levels, with statistically significant differences (*P <* 0.05). A pairwise comparison of the coagulation indicators (D-dimer, FDP and aPTT), which were statistically significant in the four groups with different imaging presentations, indicated no statistically significant difference in the pairwise comparison of aPTT across the four groups (*P* > 0.05). Pairwise comparisons of FDP between the four groups showed no statistically significant differences (*P* > 0.05) for Group B/Group C and Group C/Group D, and statistically significant differences (*P <* 0.05) in the pairwise comparisons for Group A/Group B, Group A/Group C, Group A/Group D and Group B/Group D. Pairwise comparisons of D-dimers across the four groups showed that the difference was not statistically significant (*P* > 0.05) only in the Group B/Group C comparison, with the difference statistically significant (*P <* 0.05) in all other pairwise comparisons across the groups (Fig. 1).

### Comparison of coagulation indicators in children with *Mycoplasma pneumoniae* pneumonia with different bronchoscopic manifestations

A comparison of the coagulation indicators, TT, PT and FIB, in different bronchoscopic presentations showed no statistically significant differences (*P* > 0.05). The D-dimer expression and FDP levels were higher in the mucus plug group than in the non-mucus plug group, while the aPTT levels were lower in the former than in the latter, with statistically significant differences (*P <* 0.05) (Table 3).

**Table 3:** Comparison of coagulation parameters in different bronchoscopic findings

Coagulation Indicators	Mucus plug group(n = 36)	Non-mucus plug group(n = 49)	*t*/*Z* value	*p* value
TT(s)	17.26 ± 1.29	16.98 ± 1.70	0.847	0.399
PT(s)	13.96 ± 1.32	13.63 ± 1.58	0.130	0.319
APTT(s)	28.96 ± 6.48	32.06 ± 5.63	2.350	0.021
FIB(g /L)	4.37 ± 0.90	4.19 ± 0.88	0.912	0.364
D-Dimer(mg /L)	2.95(1.20, 5.50)	0.72(0.51, 1.91)	4.278*	< 0.001
FDP(µg/L)	13.36(6.69, 26.47)	4.53(3.10, 9.04)	4.642*	< 0.001
NP,n(%)	17(47.22)	5(10.20)		< 0.001
SMPP,n(%)	36(100)	28(57.14)		< 0.001

Note: * represents the Z value; TT = thrombin time; PT = prothrombin time; APTT = activated partial thromboplastin time; FIB = Fibrinogen; FDP = fibrinogen degradation products; NP = necrotizing pneumonia; SMPP = Severe Mycoplasma pneumoniae pneumonia

### Multi-factor logistic regression analysis of the predictive value of coagulation indicators in the development of necrotising pneumonia in severe *Mycoplasma pneumoniae* pneumonia

The comparison of the coagulation indicators, TT, PT, APTT, FIB and FDP, for predicting the development of NP in SMPP indicated no statistically significant differences (*P* > 0.05). However, there was a statistically significant difference in the comparison of D-dimer expression in predicting the development of NP in SMPP (D-dimer: odds ratio [OR] = 2.470, 95% confidence interval [CI] 1.41–4.33, *P* < 0.05) (Table 4).

**Table 4:** Logistic regression analysis of coagulation parameters in patients with necrotizing pneumonia in SMPP

Coagulation Indicators	*p* value	*OR* value	95% confidence interval of *OR*
TT(s)	0.307	1.404	0.73–2.70
PT(s)	0.628	1.184	0.59–2.35
APTT(s)	0.556	0.963	0.85–1.09
FIB(g/L)	0.272	2.004	0.58–6.93
FDP(µg/L)	0.484	0.991	0.97–1.02
D-Dimer(mg/L)	0.002	2.470	1.41–4.33

Note: TT = thrombin time; PT = prothrombin time; APTT = activated partial thromboplastin time; FIB = fibrinogen; FDP = fibrinogen degradation products; SMPP: Severe Mycoplasma pneumoniae pneumonia

### Sensitivity and specificity of receiver operating characteristic curve analysis of variables associated with *Mycoplasma pneumoniae* pneumonia severity for predicting severe infection combined with necrotising pneumonia

**Fig. 2:**
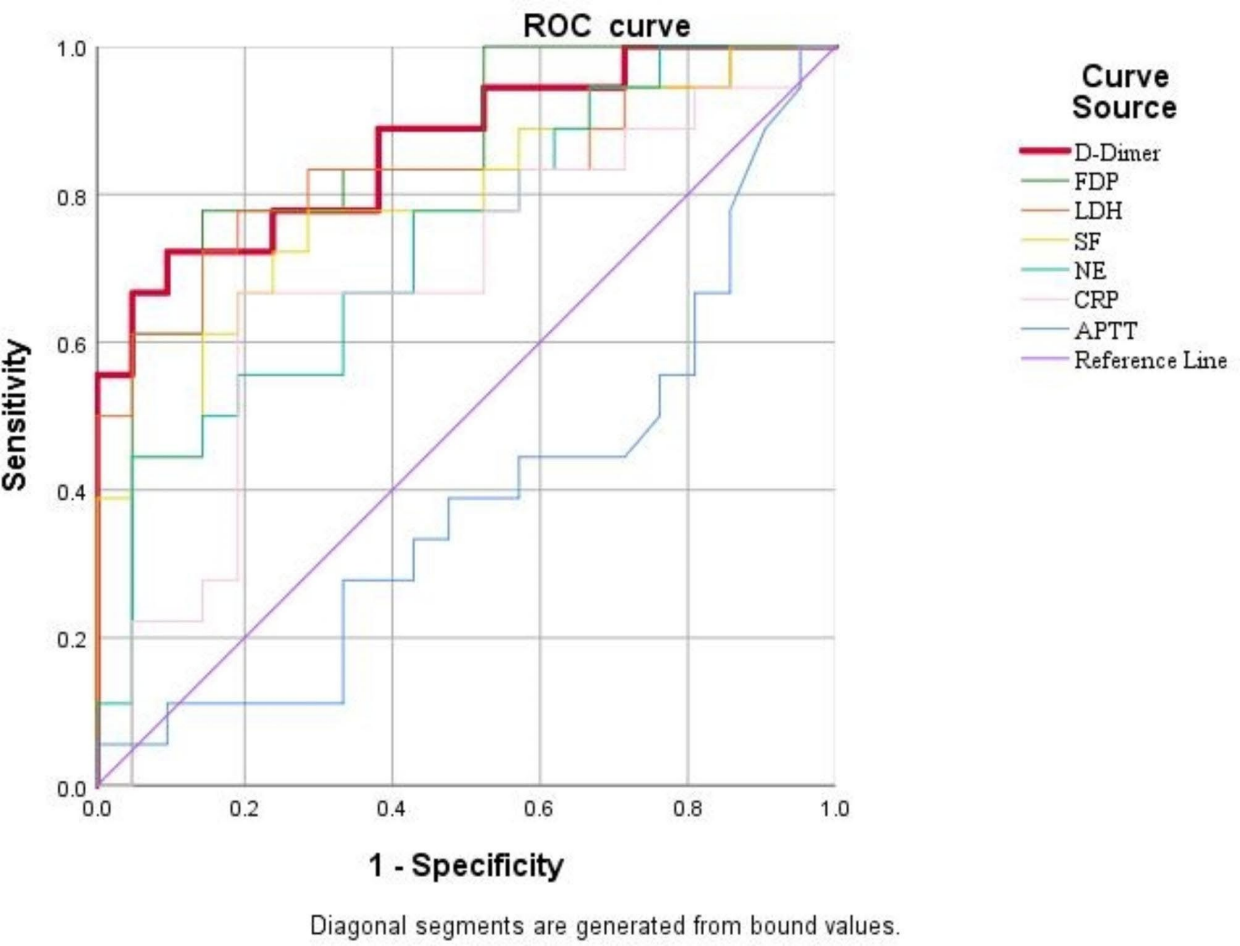
ROC curves for D-dimer, FDP, LDH, SF, NE%, CRP, APTT. Note: MMP: *Mycoplasma* pneumoniae pneumonia, FDP: fibrinogen degradation products, LDH: lactate dehydrogenase, SF: serum ferritin, NE%: Neutrophils, CRP:C-reactive protein, APTT: activated partial thromboplastin time, * p<0.05, **** p<0.0001

The ROC curve analysis of variables associated with the severity of MPP infection showed no statistically significant difference in the comparison of CRP and aPTT for predicting SMPP combined with NP (*P* > 0.05). The comparisons of D-dimer, FDP, LDH, SF and NE% levels for predicting SMPP combined with NP showed statistically significant differences (*P <* 0.05). D-dimer expression had the largest area under the ROC curve (AUC), with an AUC of 0.865 and an optimal cut-off value of 3.705 mg/L. The sensitivity and specificity for predicting SMPP combined with NP were 72.2% and 90.5%, respectively (Fig. 2).

## Discussion

In recent years, the increased resistance of MP to macrolides and severe inflammatory responses, such as direct invasive damage and immune damage caused by MP infection, has led to an increase in the incidence of severe or refractory MPP cases, as well as an increase in comorbidities and sequelae of MPP. [16] In addition to respiratory disease, MP has been associated with a large number of diseases outside the respiratory system, with up to 25% of MP respiratory infections complicated by the involvement of additional diseases, [17] although the exact pathogenesis is not yet fully understood. This study found that coagulation abnormalities were closely related to the severity of MPP in children, with elevated serum D-dimer levels (> 3.705 mg/L) being an independent predictor of MPP combined with NP in children. This result could help clinicians to accurately assess the condition, promptly identify serious pulmonary complications and provide early comprehensive treatment measures to shorten the course of the disease and improve the prognosis. Given the limited content of this study, multiple controlled studies and multicentre studies could be conducted in the future to reduce bias and further confirm the findings of this study, as well as multiple pathogenic microbiological tests to exclude other factors that may affect the test results. It is also possible to analyse the changes in coagulation in MPP in conjunction with other tests, such as thromboelastography; the underlying mechanisms could be explored to gain insight into the mechanisms of coagulation abnormalities and MPP.

Under normal conditions, the coagulation and fibrinolytic systems are in dynamic equilibrium. When coagulation occurs in the body, thrombin acts on fibrin to activate the fibrinolytic system and form D-dimers, which are not only specific markers of the fibrinolytic system but can also be used as an indicator for monitoring inflammation and serious infections. [7] The D-dimer levels are also closely associated with the inflammatory response and may reflect the coagulation status of infectious diseases. It has been found that the progression of MPP is mostly accompanied by disorders of the coagulation system and is closely related to the condition’s prognosis. [18] Another study found higher D-dimer levels in children with MPP than in healthy children and higher D-dimer levels in patients with SMPP, particularly those with SMPP with extra-pulmonary complications. [19] In the present study, the incidence of coagulation abnormalities was significantly higher in the SMPP group than in the NMPP group. The D-dimer, FIB and FDP levels were significantly higher in the SMPP group than in the NMPP group, whereas the aPTT levels were lower in the former than in the latter. More specifically, the difference in D-dimer level was the most significant between these two groups, which was similar to the results obtained in previous studies [19] and also suggests that there may be a correlation between coagulation abnormalities and the severity of the disease. This study then compared the coagulation function between groups with different levels of MPP severity and found that the differences in D-dimer, FDP and aPTT levels were statistically significant among the four groups with different imaging presentations, with the differences in D-dimer and FDP being more significant. This finding is consistent with previous studies that have found the degree of increased D-dimer levels to be positively correlated with the severity observed in lung imaging. Here, higher D-dimer levels are associated with more severe lung imaging results, manifesting as lung injury, particularly in patients with combined pulmonary necrosis or extra-pulmonary complications. [20, 21]

Necrotising pneumonia is a serious complication of SMPP in children that, if not treated promptly, can lead to serious complications, such as bronchopleural fistula, septic pneumothorax, haemoptysis, respiratory failure and septic shock, and may permanently damage lung structure or function. It is thus essential to explore biomarkers that can predict the development of NP at an early stage. Previous studies have shown that NP is associated with a severe systemic inflammatory response, and its development can be predicted early by detecting changes in peripheral blood leukocyte count, LDH, NE%, peak CRP, procalcitonin and other inflammatory markers. [22] Another study showed that a white blood cell count of > 12.3 × 10^9^/L, an NE% of > 73.9% and a D-dimer level of > 1,367.5 ng/mL are risk factors for the development of pulmonary necrosis in patients with SMPP with solid lung lobar changes. [23] In the present study, coagulation level was found to be closely related to the severity of MPP infection. By constructing a multi-factor logistic regression equation, the D-dimer level was found to be an independent risk factor for predicting NMPP combined with NP. The ROC curve analysis revealed that the D-dimer level, FDP, NE%, LDH and SF could be used as predictors of NMPP combined with NP. Among them, the D-dimer level had the largest AUC (0.865), with its predictive ability for NMPP combined with NP demonstrating the best cut-off value at 3.705 mg/L. The sensitivity and specificity of the D-dimer level for predicting the occurrence of NP in MPP were 72.2% and 90.5%, respectively. This was similar to the findings of Qian J et al. [20, 24] whose study results indicate that elevated levels of D-dimer have a high predictive value for the occurrence of pulmonary necrosis. A bronchoscopy was also performed in children with NMPP combined with solid lung lesions. By observing the bronchoscopic presentation, abnormalities in coagulation in the group with bronchoscopic mucus plug presentation were compared with those in the group without a mucus plug. The results suggest that the D-dimer and FDP levels in the mucus plug group were significantly higher than those in the non-mucus plug group, while the aPTT levels were lower in the former than in the latter, with the difference both statistically significant and consistent with the imaging results. Previous studies have concluded that bronchoscopy allows for direct visualisation of tracheobronchial mucosal lesions in children with MPP and can determine the severity and nature of bronchial obstruction. It also allows for the timely removal of airway sputum and inflammatory factors via bronchoscopic alveolar lavage, as well as for assessing the prognosis and reducing the incidence of sequelae, thus playing an important role in the diagnosis, severity assessment and identification of the intrapulmonary complications of MPP. [25–29] This study also found that SMPP is prone to bronchial lumen narrowing or atresia and bronchial mucosal erosion, in addition to bronchial mucus plug embolism. The higher the D-dimer and FDP levels, the longer the fever lasted, and the greater the risk of NP and extra-pulmonary complications and long-term sequelae developing, such as atelectasis and occlusive bronchitis. It is hypothesised that a comprehensive analysis of coagulation and other inflammatory parameters, combined with early bronchoscopy, could assist clinicians in the early identification of SMPP and RMPP.

There are some limitations to this study. First, as this is an observational study, selection bias may be present. Second, despite our best efforts to perform relevant laboratory tests, it is possible that the children may have had a combination of other pathogens, such as neo-coronavirus infection, which may also have impacted the test results. Our previous study concluded that there was no significant change in the incidence of MPP before and after the outbreak of the new coronavirus infection; however, the study was limited by a small sample size and further validation using an expanded sample size is thus needed. Third, mismatching in the distribution of the two groups of patients, with individual data missing, may also have affected the statistical results.

## Conclusion

The degree of elevated D-dimer and FDP levels was positively correlated with the severity of MPP, and serum D-dimer level (> 3.705 mg/L) could be an independent predictor of MPP combined with NP in children.

## Data Availability

The datasets generated/analyzed for this study can be found in this article.

## List of abbreviations

MPP: Mycoplasma pneumoniae pneumonia
NP: necrotising pneumonia
SMPP: severe MPP
NMPP: normal MPP
FDP: fibrinogen degeneration products
RMPP: refractory MMP
CT: computed tomography
TT: thrombin time
PT: prothrombin time
aPTT: activated partial thromboplastin time
FIB: fibrinogen
NE%: neutrophil ratio
CRP: C-reactive protein
SF: serum ferritin
LDH: lactate dehydrogenase
Ig: immunoglobulin
ROC: Receiver operating characteristic curve
AUC: area under the curve
